# Chromium Toxicity in Plants: Signaling, Mitigation, and Future Perspectives

**DOI:** 10.3390/plants12071502

**Published:** 2023-03-29

**Authors:** Sajad Ali, Rakeeb A. Mir, Anshika Tyagi, Nazia Manzar, Abhijeet Shankar Kashyap, Muntazir Mushtaq, Aamir Raina, Suvin Park, Sandhya Sharma, Zahoor A. Mir, Showkat A. Lone, Ajaz A. Bhat, Uqab Baba, Henda Mahmoudi, Hanhong Bae

**Affiliations:** 1 Department of Biotechnology, Yeungnam University, Gyeongsan 38541, Republic of Korea; 2 Department of Biotechnology, Central University of Kashmir, Ganderbal 191201, India; 3 Plant Pathology Lab, ICAR-National Bureau of Agriculturally Important Microorganisms, Maunath Bhanjan 275103, India; 4 MS Swaminathan School of Agriculture, Shoolini University, Bajhol 173229, India; 5 Mutation Breeding Laboratory, Department of Botany, Aligarh Muslim University, Aligarh 202002, India; 6 ICAR-National Institute for Plant Biotechnology, New Delhi 110012, India; 7 Centre of Research for Development, University of Kashmir, Srinagar 190006, India; 8 Govt. Degree College for Women, University of Kashmir, Baramulla 193101, India; 9 Directorate of Programs, International Center for Biosaline Agriculture, Dubai P.O. Box 14660, United Arab Emirates

**Keywords:** chromium toxicity, hormones, multiomics, priming, signaling, genome editing, breeding, synthetic biology, nano priming

## Abstract

Plants are very often confronted by different heavy metal (HM) stressors that adversely impair their growth and productivity. Among HMs, chromium (Cr) is one of the most prevalent toxic trace metals found in agricultural soils because of anthropogenic activities, lack of efficient treatment, and unregulated disposal. It has a huge detrimental impact on the physiological, biochemical, and molecular traits of crops, in addition to being carcinogenic to humans. In soil, Cr exists in different forms, including Cr (III) “trivalent” and Cr (VI) “hexavalent”, but the most pervasive and severely hazardous form to the biota is Cr (VI). Despite extensive research on the effects of Cr stress, the exact molecular mechanisms of Cr sensing, uptake, translocation, phytotoxicity, transcript processing, translation, post-translational protein modifications, as well as plant defensive responses are still largely unknown. Even though plants lack a Cr transporter system, it is efficiently accumulated and transported by other essential ion transporters, hence posing a serious challenge to the development of Cr-tolerant cultivars. In this review, we discuss Cr toxicity in plants, signaling perception, and transduction. Further, we highlight various mitigation processes for Cr toxicity in plants, such as microbial, chemical, and nano-based priming. We also discuss the biotechnological advancements in mitigating Cr toxicity in plants using plant and microbiome engineering approaches. Additionally, we also highlight the role of molecular breeding in mitigating Cr toxicity in sustainable agriculture. Finally, some conclusions are drawn along with potential directions for future research in order to better comprehend Cr signaling pathways and its mitigation in sustainable agriculture.

## 1. Introduction

Heavy metal (HM) pollution has become a major concern in sustainable agriculture due to its adverse effects on crop growth, soil health, food safety, and marketability. Numerous HMs, including arsenic (As), cadmium (Cd), chromium (Cr), cobalt (Co), lead (Pb), and mercury (Hg), cause extreme toxicity in plants when they enter agricultural soil ecosystems through anthropogenic or natural activities [1]. Rapid anthropogenic activities such as mining, industrialization, and agricultural chemical practices have caused a notable rise in the bioaccumulation and biomagnification of HMs, which has a negative impact on the food chain and the environment [2]. Among HMs, Cr is potentially hazardous and serves no vital role in plant metabolism. It is the second most frequent metal pollutant in soil, groundwater, and sediment, and it poses a serious environmental risk [3]. The International Agency for Research on Cancer [4] and the National Toxicology Program both rank Cr as the number one carcinogen raising serious concerns for human health. Cr is found in soil in a variety of chemical forms, mainly chromite [Cr (III)] and chromate [Cr (VI)], both exhibiting significantly distinct biogeochemical properties [5]. Disentangling the mechanism underlying Cr plant toxicity has been extremely difficult due to its complicated electrical chemistry. Although Cr (III) is a necessary trace element from the perspective of animal and human health, but not for plants [6]. Since Cr is non-essential and a toxic element to plants, no Cr-specific transporters or channels have been identified in plants so far. Instead, in the majority of plant species, Cr is transported by some essential element transporters. After Cr exposure to plants, numerous physiological, morphological, and metabolic traits are adversely affected that ultimately lead to plant death. For instance, Cr toxicity affects plant development, nutrient absorption, and photosynthesis while also increasing the production of reactive oxygen species (ROS) and altering antioxidant activities [7]. Cr toxicity has a major impact on our sustainable agriculture and food security as it affects most of the agriculturally important crops, such as pulses, cereals, vegetables, etc. [7,8,9,10]. Furthermore, Cr buildup in crops from contaminated soils poses serious health concerns to humans and animals. Therefore, a thorough understanding of Cr’s biogeochemical activity in soil, its toxicity to plants, and the development of a long-lasting remediation toolkit are necessary for its mitigation, which will be beneficial from the perspectives of both sustainable agriculture and human health. This review focuses on Cr toxicity in plants, the signaling responses to Cr stress, and the role of microbial, chemical, and nano priming for mitigating Cr toxicity in plants. Additionally, we also discuss breeding and biotechnological advancements in mitigating Cr toxicity in plants with a focus on genome editing. Additionally, knowing the molecular basis of Cr–plant interactions and its biogeochemical chemistry can offer fresh perspectives on how it can be mitigated through genetic, chemical, and microbiological means, which would boost crop yield and agricultural sustainability.

## 2. Sources of Chromium

**Figure 1:**
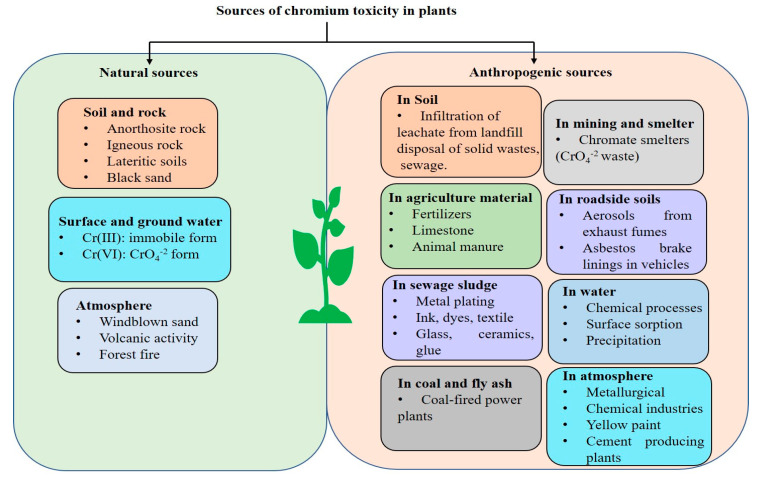
Sources of Cr contamination in agricultural soils and other environments. In this figure, we have highlighted both natural and anthropogenic sources of Cr in agricultural soils.

Cr contamination has become a major problem in the environment due to its high concentration in various agricultural and industrial activities [11]. The estimated tolerable limit of Cr in soil from the perspective of the protection of human and environmental health is around 64 mg/kg. The element Cr was originally identified in 1797 as a component of the pigment-grade mineral crocoite (PbCrO_4_). Due to its usage in numerous industrial and agricultural processes, a significant amount of Cr is mined or generated annually, which also contributes to its contamination in soil and water. For example, significant levels of Cr are released in different environmental sections through industrial activities such as cement and steel plants, leather factories, electroplating, paints and dyes, metal plating, timber processing, paper production, and leaching processes [12]. Additionally, the fallout of ash from burning coal or municipal trash for energy generation as well as fertilizer industries have also significantly increased the Cr (VI) content in soil and water [13]. Natural sources of Cr include rocks, volcanic dust, gases, soil, animals, and plants. Typically, Cr is strongly coupled with primary rock-derived phases and well-crystallized iron oxides [14]. Chromite (FeCr_2_O_4_) is a naturally occurring form of Cr in ultramafic rocks or serpentine as a part of different minerals, such as tarapacaite, crocoite, vauquelinite, and bentorite [15]. Furthermore, due to natural leaching from rocks and topsoil, significant levels of Cr also enter water bodies. Further, we have shown different sources of Cr contamination in soils and crops in Figure 1.

## 3. Chromium Uptake and Translocation

Plants are capable of absorbing different forms of Cr, but the mechanism by which they do so is yet unknown. There has not been any information on a specific mechanism for plant Cr uptake because Cr does not play any vital role in plant metabolism. However, plants mostly take up Cr through specialized transporters for the ion absorption required for different metabolic processes. In plants, the rate of Cr uptake, accumulation, and translocation vary greatly due to its metal speciation, which determines its overall toxic effects. Cervantes et al. [16] described the active mechanism for Cr (VI) transport, which involved transporters of essential anions such as sulfate. It has been observed that Cr competes with sulfur (S), phosphorous (P), and iron (Fe) for carrier binding during transportation [17]. Owing to the structural similarities between Cr (VI), phosphate, and sulfate, plants actively absorb Cr (VI) typically via phosphate or sulfate transporters [18,19]. The SULTR gene family (Sulfate Transporter), also called H^+^/SO_4_^2−^ transporters, have been discovered in all photosynthetic species that have been examined thus far. These are known to be potential targets for regulating Cr (VI) flow in plants. In addition to competing for sulfate transporters, Cr (VI) can also do so for the enzymes involved in the sulfate assimilation pathway, which lowers the production of cysteine (Cys) and methionine and results in the incorrect translation of crucial proteins, leading to S starvation. 

In barley, separate uptake mechanisms for Cr (VI) and Cr (III) have been discovered. For instance, using metabolic inhibitors decreased the absorption of Cr (VI) but had no effect on the uptake of Cr (III), indicating that Cr (VI) uptake is energy-dependent while that of Cr (III) is energy-independent [20]. Previous studies have reported that sulfur accumulators such as Brassica species absorb high levels of Cr [21], indicating that S uptake and translocation mechanisms are used to move Cr from root to shoot [16]. Similarly, Fe accumulators such as *Spinacia oleracea* and *Brassica rapa* can also absorb higher levels of Cr and translocate it in their aerial tissues [22]. The majority of investigations have shown an excessive buildup of Cr in roots, and its immobilization in the vacuoles of plant root cells is primarily responsible for its bioaccumulation. In roots, Cr prevents cell division and inhibits root length, which limits their ability to absorb water and nutrients, thereby restricting shoot growth. It has been determined that Cr is primarily transferred through the plant xylem after absorption [7]. In plants, Cr (VI) is transported through the endoderm and reduced to Cr (III), which is subsequently preserved in the cells of the root cortex. The majority of research has shown that Cr accumulates excessively in roots, and it is thought that this bioaccumulation is mostly caused by its immobilization in root cell vacuoles [23]. An earlier investigation into the bioaccumulation of Cr in *Brassica juncea* under CrCl_3_ stress revealed that when exposure increased, the amount of Cr in the cell wall, nuclei, mitochondria, and plastids likewise increased significantly [24]. The sequestration of Cr mainly occurs in plant roots as it is the primary organ where it absorbed from the soil. Additionally, Cr is considered to be the least transportable element among the heavy metals in the plant roots [25]. 

According to Shanker et al. [20], roots can have a Cr concentration 100 times higher than shoots. It is likely that the formation of insoluble Cr compounds inside plants is due to the increased sequestration of Cr in plant roots. Nevertheless, many metal transporter gene families, such as HMA (heavy metal ATPase), ATP binding cassette (ABC) superfamily, CDF (cation diffusion facilitator), NRAMP (natural resistance-associated macrophage protein), and ZIP (ZRT, IRT-like protein) have been identified that play crucial roles in the transportation of different metals from root to shoot [26]. However, we are still far from having a thorough understanding of these transporter families with regard to Cr in plants, despite their significant roles in metal absorption, transportation, sequestration, and tolerance. In addition to sulphate transporters, new players of Cr transportation in model and other crop systems need to be further studied and identified. This will help us to understand where and how Cr regulates different signaling pathways and will facilitate the development of crops that are tolerant to Cr in the future.

## 4. Impact of Chromium Toxicity on Different Plant Traits

**Figure 2:**
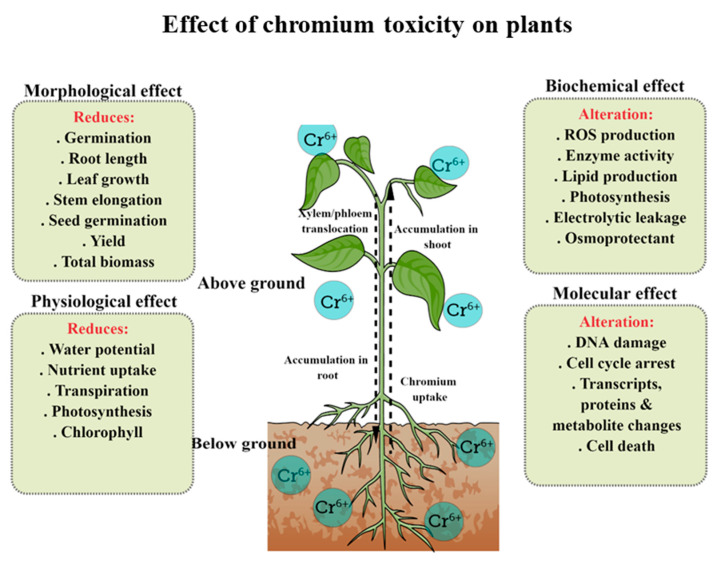
Effect of Cr toxicity (in the form of Cr^6+^ or CrO_4_^2−^) on various morphological, physiological, and biochemical traits in plants.

In plants, Cr toxicity causes a detrimental effect on various physiological, biochemical, and molecular traits, thus stunting growth and reducing overall yield production. According to Dotaniya et al. [27], higher Cr accumulation in plants significantly affects seed germination and slows down root and shoot growth rates, which has an impact on the overall biomass and yield. Many studies have reported that high Cr accumulation in plants affects the chlorophyll (Chl) content (Chl a, b, and total), leading to the inhibition of photosynthesis. Previous studies have shown that an excessive amount of Cr deposited in plant tissues inhibits the cell cycle, water and mineral balance, enzyme activity, nitrogen assimilation, the antioxidant system, and other key metabolic processes [28]. Moreover, Cr accumulation triggers the generation of ROS, which causes oxidative damage [29]. For instance, ROS buildup results in the peroxidation of membrane lipids, which disrupts membrane function and structure as well as causing the oxidation of proteins and nucleic acids, resulting in cellular component damage and eventually cell death [30]. Additionally, increased ROS prevents plants from responding biochemically, which alters their morphology and architecture [31]. The cytotoxic and genotoxic effects of Cr have been reviewed in various plants, viz*., Vigna radiata*, *Nicotiana tabacum*, *B. juncea*, *Cicer arietinum*, *Brassica napus, Brassica oleracea*, *Sorghum bicolour,* and *Zea mays* [32]. Further, we have summarized the effects of Cr on different crop traits in Figure 2.

## 5. Molecular Mechanisms and Signal Transduction in Regulating Chromium Stress in Plants

Because plants cannot escape environmental pressures such as metal pollution, these challenges have driven the evolution of numerous mechanisms to efficiently detect, react, and ultimately adapt to these pressures. Likewise, plants also use multidimensional defense responses against Cr toxicity via complexation by organic ligands, vacuolar compartmentalization, and activation of the antioxidative system. These defense responses are modulated or regulated by an intricate signaling cascade that takes place in different cellular components. Although a plethora of studies have highlighted how Cr induces the oxidative stress that leads to an array of detrimental effects on its cellular system, the exact molecular mechanisms of Cr translocation, accumulation, phytotoxicity, and plant defensive responses are still largely unknown. For plants to adapt to biotic and abiotic stressors, signal perception, transduction, and post-translational regulation are crucial. The initial perception of stressors involves different sensors, such as cell wall receptors and ion channels, as well as signaling molecules, including calcium, ROS, hormones, protein kinases, and transcriptional factors, that play key roles in downstream signaling cascades. The roles of the above signaling players have been well addressed during biotic and many abiotic stressors. However, their roles in heavy metal stress, including Cr, are largely known.

The molecular dynamics of Cr signaling from the exterior (cell wall) and interior (plasma membrane and cytosol) are not fully understood and many knowledge gaps are still remaining. However, with the advent of multiomics, a few studies have recently reported transcriptional, translational, and metabolic reprogramming in various plant systems after Cr exposure, thus providing novel insight into Cr perception and signal transduction. For example, in rice plants, Cr (VI) triggered ROS and Ca^2+^ production followed by activation of NADPH oxidase and calcium-dependent protein kinase, all of which are critical for downstream signaling cascades [33]. There is mounting evidence that calcium and ROS signaling systems interact reciprocally, with important ramifications for optimizing cellular signaling networks. They also identified many transcriptional factors involved in Cr signaling cascades, such as WRKY and AP2/ERF TF genes, which added to the notion of their role in defense against metal stress. Similarly, many phosphate kinase genes (PP2C-A, PP2C-D, and PP2C-F) were identified in response to Cr (VI) stress, which further provided evidence that these might be involved in regulating various signaling cascades during Cr stress. Earlier gene expression profiling of rice plants under Cr stress revealed the inactivation of gibberellic acid-related pathways and stimulation of ethylene (ET), abscisic acid (ABA)-, and jasmonate-mediated signaling cascades. This provided novel insight into the role of different hormones during Cr stress [33]. Another study reported that transcriptome profiling of rice plants after Cr (VI) exposure showed a distinct gene expression profile. For example, genes involved in membrane transport and signal transduction, xenobiotics, amino acid metabolism, and biosynthesis of secondary metabolites were upregulated, whereas genes related to cell growth and energy metabolism were downregulated. Huang et al. [34] found that Cr (VI) induced an array of genes related to ROS, calcium, MAPKs, and CDPK-like kinases, all of which are key players in perception and signal transduction pathways. Similarly, various miRNA were identified in tobacco plants that were distinctly regulated during Cr (VI) stress [35].

**Figure 3:**
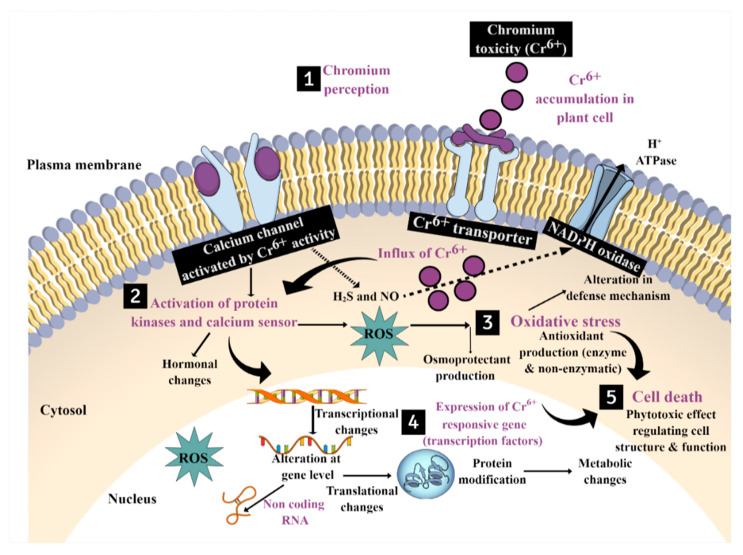
A hypothetical model showing Cr perception and signal transduction in plants. Plants have different types of sensors, such as receptor-like kinases and channels, that can take part in early Cr perception. Following Cr sensing, an ROS burst and calcium waves will occur, which can be sensed by different sensors, such as kinases or calcineurin B-like protein (CBL)-CBL interacting protein kinase (CIPK) and calmodulins (CaMs)/calmodulin-like proteins (CMLs), which can lead to significant transcriptional and translational reprogramming in several intracellular compartments, as depicted in the figure. The Cr (Vi) transporter in this instance is a sulphate or phosphate transporter, which could ease its entry into the root cells. We also highlight the roles of various molecules, including hormones, nitric oxide (NO), hydrogen sulfide (H_2_S), and antioxidants, in Cr-mediated signaling.

On the other hand, a proteomic perspective has also been used to identify differential proteins during Cr stress. For example, 64 proteins were successfully identified in rice seedlings that were related to several cellular processes, viz., cell wall synthesis, electron transport, primary metabolism, energy production, and detoxification [36]. In the last 10 years, a number of studies have been published on the effects of HMs on the metabolome of both model plants and cultivated cultivars. Similarly, a metabolomics study of rice plants after Cr exposure showed a significant accumulation of proline and ornithine, which could be involved in the defense response of rice plants against oxidative stress during Cr exposure [37]. These studies further highlight the importance of omics tools in identifying various key players in Cr signal perception and transduction. However, the integration of multiomics along with gene knock out studies is further required to determine the roles of different genes or other key signaling players in Cr signaling that will provide novel insight for the development of Cr-tolerant crop cultivars. In this review, based on the available data, we have presented a model describing Cr signaling in plants, as shown in Figure 3. We also highlight some of the important players in the initial and downstream signaling cascades that might be involved in Cr signal perception and transduction. However, more comprehensive studies are required to fully understand the molecular dynamics of Cr signaling in plants, such as the roles of cell wall sensors, plasma membrane channels, and intracellular signaling cascades in different compartments after Cr exposure.

## 6. Mitigation of Chromium Toxicity in Sustainable Agriculture

In recent years, the levels of Cr contaminants in various ecosystems have dramatically increased due to growing urbanization and industry, which has become a serious concern across the globe. Given the detrimental effects of Cr contamination on plants and human health, it is critically important to look into quick, efficient and cost effective approaches to remove Cr from the soil and other environmental locations. Additionally, Cr poses a potential risk because it is not degradable and will remain in the soil for years. In this review, we systematically discuss various mitigation approaches against Cr toxicity in sustainable agriculture. Firstly, we discuss the role of microbes in mitigating or reducing Cr toxicity in plants since microbes act as metal cleaners owing to their incredible metal-tolerant properties. Secondly, we focus on the role of chemical priming in mitigating Cr toxicity. There have been numerous reports on the role of various chemicals (hormones, NO, H_2_S, polyamines, compatible solutes, ions, etc.) on mitigating HM toxicity in plants. Thirdly, we discuss the role of nanoparticle-based priming, which has become one of the major frontiers against HM toxicity in sustainable agriculture. Finally, we focus on the role of biotechnological tools in mitigating Cr toxicity. The use of biotechnological methods is becoming more and more common in HM remediation since they are frequently seen as a viable technique for the final treatment of contaminated sediments. With the advancement of genome editing, this strategy appears to be the most effective technique for the development of Cr-tolerant crop cultivars in sustainable agriculture. Additionally, integrated Cr remedial technologies can be very useful for in-situ operations in both developed and emerging nations where urbanization, agriculture, and industry are passing on a legacy of environmental degradation.

### 6.1. Microbe-Mediated Mitigation for Chromium Toxicity

**Figure 4:**
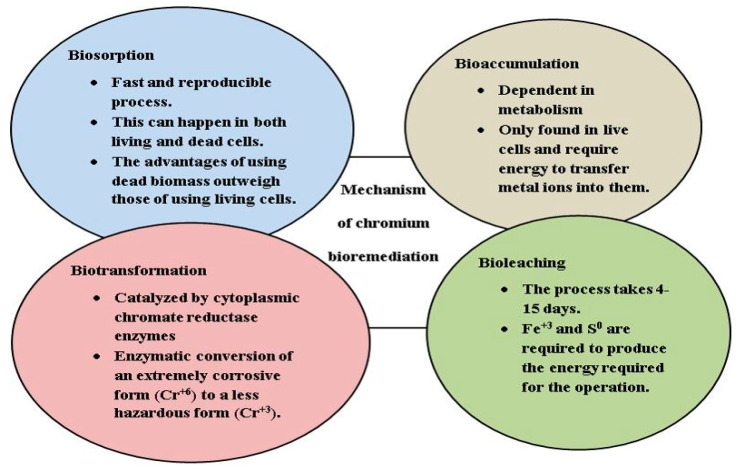
Microbe-mediated mitigation of Cr (VI) polluted environment. Microbes evolve different process such as biosorption, bioaccumulation, biotransformation, and bioleaching to remove Cr from contaminated soils. Microbes have incredible resistance mechanisms, such as extracellular and intracellular sequestration, extracellular barriers, active transport of metal ions, and enzymatic detoxification, which makes them the best remedial tool kit for heavy metal remediation.

Many microorganisms have a remarkable capacity to adapt and colonize toxic HM-polluted habitats that are unsuitable for higher organisms. Absorption, adsorption, methylation, oxidation, and reduction are just some of the ways that these microbes have developed to defend themselves against HM contamination. The conversion of Cr (VI) to less harmful Cr (III) is a crucial step in the cleanup of Cr (VI)-affected areas. Traditional methods for Cr (VI)-contaminated groundwater and soil involve pumping and excavating the contaminated material, then adding chemical reductants, which results in the sedimentation and precipitation of the reduced Cr [38]. These methods have a number of disadvantages in addition to being costly and energy-intensive. In this context, bioremediation of Cr has recently gained popularity as a safe, ecofriendly, and cost-effective alternative to standard physico-chemical approaches. However, the availability of effective microbial consortia that can more effectively reduce or eliminate Cr (VI) is necessary for the bioremediation of Cr (VI)-contaminated soil and water. Interestingly, microorganisms can thrive in environments with high levels of HM contamination. Most bacteria that resist Cr (VI) are extracted from tanning wastewater and are subsequently employed to treat environmental pollution [39]. Despite the fact that microbial remediation technology has been studied extensively, there are still numerous issues to be resolved [40]. Many studies are limited to the laboratory and focus on remediating contaminated water sources, with microbiological remediation strategies in soil getting minimal attention. To address HM pollution in groundwater, these microbial strains are grown from industrial effluents or microbes are screened from the soil. The appropriate concentration needed for microbiological reduction is much lower than that needed for physical and chemical components; therefore improving bioremediation effectiveness is a major challenge. Microbial inoculation in a Cr (VI)-polluted environment can successfully remove this HM by biological adsorption, chelating agent production, autotrophic leaching, reducing agent, and other mechanisms [41], as shown in Figure 4.

In general, microorganisms can use enzymatic or chemical pathways for Cr reduction, either separately or in combination. Soluble chromate reductases are found in bacteria such as *Pseudomonas*, *Bacillus*, *Leucobacter* spp., and *Streptomyces* (using NADPH or NADH as cofactors to reduce Cr). Microbacterium strains that can withstand or resist HMs such as Cr, As, Ni, and Cd have been identified as bioremediation possibilities, as shown in Table 1 [42]. The use of native, non-hazardous strains is one of the primary benefits associated with the utilization of bacterial Cr (VI) reduction since it does not demand a high-energy input nor does it call for the utilization of harmful chemical reagents. Extracellular precipitation is a distinctive feature of bacterial interaction with Cr. Precipitation is viewed as a detoxification process because insoluble metallic complexes are usually less hazardous than ionic versions [43]. In sulfate-reducing bacteria and *Clostridium* [44], Cr precipitation has been commonly seen. With the addition of 8.0% NaCl, enzymatic reduction of Cr (VI) was seen in *Halomonas sp*. strain TA-04, which was isolated from contaminated marine sediments, giving novel clues into metal reduction in halophilic environments [45]. Many bacteria have been discovered to be capable of converting Cr (VI) to Cr (III) under a variety of conditions, as shown in Table 1.

Certain microorganisms are resistant to Cr and can convert Cr (VI) to Cr (III), as was originally seen for *Pseudomonas* spp. [73,74]. The ability of bacteria to reduce Cr (VI) to Cr (III) as a mechanism of resistance to Cr (VI) has been described in a number of bacteria. *Escherichia coli* [75], *Bacillus firmus* KUCr1 [76], *Pantoea stewartii* ASI11 [77], *Cellulosi microbium* sp. [78], and *Pseudomonas aeruginosa* CCTCC AB93066 [63] are among the bacteria shown to be capable of reducing Cr (VI). There has been evidence of the mechanisms of chromate resistance, especially at the level of bacterial cells. Because the majority of bioreduction processes are enzyme-mediated, changes in temperature and pH can have a significant impact on protein folding, the ionization rate, and enzyme activity. The concentration and contact time of heavy metals affect their absorption by microbes. This metabolism-dependent mechanism occurs only in living cells and needs the use of energy to move Cr (VI) into the cells. The efflux of chromate ions from the cell cytoplasm and reduction of Cr (VI) to Cr (III) is probably one of the best mechanisms. Microorganisms can decrease Cr (VI) to Cr (III) in both aerobic and anaerobic environments. The bio-reduction of Cr (VI) that can be obtained directly as a result of microbial metabolism can be observed in aerobic environments [79]. Using *Arthrobacter* spp. for bioremediation of trash containing Cr (VI) is an excellent option. *Arthrobacter* spp. tolerated Cr (VI) at 100 mg/mL in a salt-free medium supplemented with 0.5% glucose and was able to grow in a liquid media at this concentration, reducing Cr (VI) up to 50 g/mL. Cr (VI) reduction was shown to be mostly linked to the cell’s soluble protein fraction, as demonstrated by permeabilized (treated with toluene or Triton X100) cells and crude extracts [80]. *Pediococcus pentosaceus* and *S. aureus* (2000 mg/L) and *Streptomyces* sp. CG52 (500 mg/L) are two examples of Gram-positive bacteria that are Cr (VI)-tolerant [81]. Many bacterial whole genomes have been sequenced in recent years in order to find loci implicated in metal resistance, especially Cr reduction and resistance. ChrR and yieF are implicated in the mechanism of chromate reduction, according to genomic annotations [82]. In LB agar medium, bacterial strain UT8 (MN932132) is resistant to greater doses of chromate. Cr (III) adsorption by *Phormidium laminosum* heat-dried biomass has been reported. At lower pH values (3.5 to 5.5), the binding of Cr (III) by the microalgae was boosted, while that of Cr (VI) was favoured at pH 2.0 or lower [83]. For bioremoval, the optimum pH is dependent on the oxidation state of Cr, as shown by these results in which Cr (VI) to Cr (III) reduction was found to occur with a significant rise in temperature from 25 to 55 °C [84]. Fungi are microbes that are employed as biosorbents to remove heavy metals because of their large biomass yields. Fungi, because of their versatility, ability to adapt to hard conditions, and ability to endure high concentrations of hexavalent chromium [85], can tolerate toxic concentrations of Cr (VI) beyond 10,000 mg/L. Higher metal ion concentrations rarely affect fungi [86]. A chemical interaction with functional groups on cell surface proteins was required for Cr (VI) to be adsorbed on the cell surface of fungi [87]. Chemical components in proteins, lipids, and polysaccharides such as galactosamine and chitin/glycans along with different functional groups, including carboxyl groups (-COOH), polyphosphates (PO_4_^3−^), amines (-NH_2_), sulfur groups (-SH), and hydroxide groups (-OH), are responsible for the binding of hexavalent chromium to the fungal cell [88]. Chromate sensitivity in filamentous fungi and yeasts as well as yeast chromate reduction are among the well-studied interactions between Cr and fungi.

### 6.2. Chemical Priming of Plants to Alleviate Chromium Toxicity

Chemical priming is one of the best approaches to enhance the tolerance of both cultivated and non-cultivated plant species against a wide range of stress factors. Nevertheless, to date, very few reports have backed this commitment in connection to its role in the stress tolerance of plants against abiotic stress factors [89,90,91]. Against the poor attention paid to chemical priming, we present key reports and findings to discuss updated data on chemical priming and its critical role in circumventing Cr toxicity. The Cr produced because of metallurgical processes and effluent discharges used in the tanning and preservation of wood is extremely toxic to plants [20]. Cr exists in different stable forms such as trivalent Cr (III) and hexavalent Cr (VI) species, with the latter being most stable and toxic to plant metabolism and growth [92]. The toxicity of Cr leads to the production of ROS, such as hydroxyl radicals (•OH), hydrogen peroxide (H_2_O_2_), and superoxide anions (O_2_•), which lead to several serious complications in plants, including lipid peroxidation and inhibition of enzyme activities, growth retardation, degradation of photosynthetic pigments, and chromosomal aberrations.

Hence, to minimize the detrimental effects of chromium in plants, scientists have devised diverse strategies, such as chelation, detoxification, and subcellular compartmentalization of Cr, using both biological as well as chemical methods. Recently, several reports have suggested that Cr toxicity can be alleviated by exogenous application of ABA, glutathione, Cys and sulfur, and melatonin [93,94,95]. For instance, it was reported that metallothioneins (MTs) have emerged as important ligands to chelate and detoxify heavy metal ions such as Cr in plants [96]. Way back, Chen et al. [97] reported that under salinity and drought stress, genes such as metallothionein protein (BnMP1) and metallothionein-like (LSC54) for MT were upregulated in *B. napus*, proving a lead role of MTs in stress tolerance. Reports suggest that exogenous application of H_2_S led to enhanced expression of MT genes in plants, thus providing a platform for chemical priming of plants to overcome Cr toxicity [98,99]. For instance, Mustafa et al. [100] reported that exogenous application of H_2_S helped to overcome the toxic effects of Cr (VI) in *B. napus* by enhancing the activity of antioxidant enzymes, decreasing lipid peroxidation, and increasing the thiol and chlorophyll content. Similarly, it was reported that exogenous application of 5-amenolevulinic acid (ALA) enhanced the growth and metabolism of plants and decreased the concentration of Cr in *B. napus* under Cr toxicity.

NO was found to trigger spermine in order to reduce the accumulation of Cr in rice plants in addition to its role in increasing carbon assimilation and reducing ROS-mediated damage [101]. Moreover, it was reported that taurine aided in the protection against lipid peroxidation in membranes and ROS scavenging to promote plant growth [102]. This amino acid was reported to enhance the growth of wheat plants by reducing the oxidative damage under Cr toxicity stress [103]. These results also showed that taurine triggered an increase in the concentrations of nutrients and secondary metabolites (phenolics and flavonoids) to alleviate Cr toxicity. Ahmad et al. [104] reported that taurine administration improved the accumulation of proline to enhance tolerance to boron (B) and Cr and also aided in the regulation of metabolic activities. Another chemical, H_2_S, was found to play a profound role in enhancing tolerance to Cr, aluminum, boron, and copper toxicity in addition to its role in drought stress tolerance [105,106,107]. Similarly, Cr toxicity was circumvented in barley by exogenous supplementation of H_2_S through its effect on enhancing growth via the upregulation of photosynthetic machinery [108]. The supplementation of H_2_S was found to trigger the generation of Cr^6+^-binding peptides such as metallothioneins and phytochelatins to compartmentalize Cr^6+^ to insensitive regions in *Arabidopsis* [109].

Recently, it was reported that the exogenous application of glutathione decreased the translocation, absorption, and chelation of Cr in soybean, hence improving plant biomass by adjusting the soluble proline and phenol content [110]. These compounds are reported to aid in the removal of ROS under stress conditions [111]. In addition, the accumulation and detoxification of Cr helped to enhance the plant’s physiological activities upon administration of exogenous glutathione under Cr toxicity [112]. The mechanism behind the alleviation of Cr toxicity lies in the formation of Cr–GSH complexes due to the presence of the thiol group (-SH), hence reducing free Cr in plants [113]. Moreover, glutathione was reported to neutralize the ROS generated by Cr toxicity via the formation of the ascorbate–glutathione cycle (ASA–GSH cycle) [114]. In conclusion, scientists validated the role of glutathione in considerably maintaining the chlorophyll content of plant leaves by decreasing Cr toxicity in plants [115,116].

Previous studies have reported that exogenous foliar administration of mannitol (M) to wheat plants enhanced the tolerance to Cr toxicity by decreasing Cr uptake and translocation, increasing the activity of antioxidant enzymes, and enhancing the concentration of photosynthetic pigments in plants [117]. Menadione sodium bisulfite (MSB) is another chemical used in priming plants to circumvent Cr toxicity due to its redox properties augmenting the plant’s physiological properties. MSB has been reported to reduce the levels of Cr in the aerial parts of plants, enhance the antioxidant systems, and decrease oxidative damage in plants [118]. Moreover, H_2_S reportedly alleviated Cr toxicity in barley to mediate Cr tolerance [107]. All of these reports strongly suggest that chemical priming may aid in alleviating Cr toxicity in crop plants and is a leading approach to enhance the yield and productivity of crops in the future.

Phytohormones such as auxins (IAA), brassinosteroids (BRs), ABA, cytokinins (CK), gibberellins (GA), jasmonic acid (JA), and salicylic acid (SA) are another series of biomolecules that have been employed to enhance the tolerance of crop plants against a wide range of biotic as well as abiotic stressors, including Cr, to maintain proper metabolism and physiology [119,120]. For instance, Mumtaz et al. [119] reported that 24-epibrassinolide induced commendable enhancement in the growth, physiology, and upregulation of defense systems in pepper plants under Cr (VI) stress. Cr stress was also mitigated by the application of the polyamine brassinosteroid to maintain phytochemical and physiological attributes in *Raphanus sativus* L. [121]. Additionally, it was reported that a reduction in oxidative stress was observed in *Pisum sativum* L. upon administration of indole acetic acid (IAA) on seedlings under chromium stress conditions [122]. The alleviation of Cr (VI) stress was accomplished by the application of ET and H_2_S in black bean and mung bean crop plants [123]. In this study, it was observed that H_2_S impaired ET signaling to reduce the negative effects of Cr stress. JA is another plant hormone found to prime the alleviation of chromium stress by decreasing chromium uptake, thus enhancing the regulation of glyoxalase and the oxidative defense system in choysum (*Brassica parachinensis* L.) [124]. Similarly, JA application in *P. sativum* L. seedlings was found to be associated with the regulation of other hormones and increased the uptake of mineral ions such as calcium (Ca^2+^), which was linked to detoxification under Cr toxicity conditions [125]. SA is a critical phytohormone useful for the induction of defense mechanisms against a wide range of abiotic stress factors [126,127]. This hormone ameliorates Cr toxicity by regulating ion homeostasis, the ultrastructure of cells, and also the modulation of the antioxidant defense system [128]. This brief account of phytohormones displays considerable evidence of their role in alleviating Cr toxicity in plants. Hence, it must be concluded that phytohormones can be substantially employed as a possible strategy to circumvent the toxic effects of Cr in plants.

### 6.3. Nano-Priming as Pilot Strategy to Alleviate Chromium Toxicity in Plants

Currently nanotechnology is at the forefront of attaining sustainable development of the agricultural sector through its diverse tools, such as nanosensors, nanopesticides, and nanofertilizers [129,130,131]. Evidently, nanoparticles have been employed to positively regulate the development and growth of plants and withstand the challenges of stress factors [132,133]. A large number of reports have validated the role of nanoparticles in priming seeds for speedy germination, thus leading to improved growth and tolerance to stressors and obtaining higher yield and growth [134,135]. For instance, the germination of *Festuca ovina* under drought stress was stimulated by the nanopriming approach using silver nanoparticles at concentrations ranging from 25% to 75% [136]. Similarly, zinc oxide nanoparticle (ZnONP)-based priming was utilized to enhance the resilience of rapeseed (*B. napus* L.), thereby enhancing germination under salinity stress conditions [137]. Moreover, silver nanoparticles (AgNPs, 1 mg/L) were utilized as priming agents to enhance seed germination in wheat and help to reverse the effects of salt stress in wheat plants [138]. Increased germination rates and decreased germination times were reported in hopbush (*Dodonaea viscosa* L.) seeds when supplemented with multi-walled carbon nanotubes (MWCNTs) [139]. Seed priming is another approach to enhance the stress tolerance in plants by allowing partial hydration with chemicals to amplify the different metabolic processes [136]. Nanopriming of seeds by zinc oxide significantly enhanced seed germination [136]. On the other hand, use of AgNPs with fenugreek (*Trigonella foenum-graecum*) seeds helped to promote seed germination [140]. It was reported that indole acetic acid (IAA) and silicon nanoparticles (SiNPs) in combination and alone primed rice seedlings to enhance tolerance to Cr toxicity [141]. In combination with *Staphylococcus aureus*, ZnONPs were shown to mediate the alleviation of Cr toxicity in wheat plants, thus enhancing the defense system, growth, and physiology [142]. Similarly, the application of ZnONPs enhanced the activities of CAT, APX, SOD, and POD in mustard plants under Cr toxicity conditions [139,142]. Further, we have summarized the roles of chemical priming in mitigating Cr stress in plants in Table 2.

### 6.4. Biotechnological Approaches for Mitigating Chromium Stress in Plants

Finding effective, long-lasting, and affordable solutions to remove Cr contamination from contaminated places is critical due to the negative impact of Cr on both humans and plants. In this regard, integrating multiomics approaches to decipher the molecular mechanisms of host–Cr interactions and their signaling and thus identify potential target genes can be further utilized for the development of Cr-resistant cultivars via genetic engineering. In the last decade, the advent of high-throughput ‘omics’ technologies has generated extensive information on the regulatory mechanisms of plant resistance to various biotic and abiotic stresses [156,157,158]. Similarly, omics studies and the integration of bioinformatics resources have helped in understanding the molecular mechanisms regulating heavy metal accumulation in plants and the responses of plants to these stresses. However, there are few studies on Cr–plant interactions, which leads to huge knowledge gaps. In a transcriptomic study on pepper plants, it was shown that Cr inhibited several metabolic and biochemical pathways in ZS 758, including lipid biosynthesis, stilbenoid, diarlyheptanoid, and gingerol biosynthesis, carbohydrate metabolism, and glutathione metabolism, while the ribosome and glucosinolate biosynthesis pathways were observed to be affected in Zheda-622. On the contrary, vitamin metabolism and amino acid biosynthesis were induced in the studied crop varieties. Cr has also been shown to positively influence certain transcription factors regulating different enzymes, such as hydralases, phosphatases, pyrophosphatase, and oxidoreductases. Enzymes with antioxidant activity have also been shown to be positively regulated by Cr. In a study by Goupil et al. [159], several heat shock protein genes, including Hsp90-1, were found to be upregulated in tomatoes grown under Cr stress conditions. In addition, Gill et al. [160] identified a novel Cr-responsive protein (CL2535.Contig1_All). Differential proteomics tools, such as 2-dimensional electrophoresis coupled with the MALDI-TOF, have been used to identify DEPs (differentially expressed proteins) in different plants grown under Cr (VI) stress. The data revealed that transcript levels of ATP synthase RuBisCO and coproporphyrinogen III oxidase (CPO) were significantly increased by Cr (VI) [161] in a study exploring the impact of Cr (III, VI) in pollen germination in kiwi. Comparative proteomics revealed that proteins involved in maintaining homeostasis, lipid synthesis, and the antioxidant defense system were markedly upregulated under conditions of Cr exposure and those involved in the mitochondrial oxidative phosphorylation process were significantly reduced, resulting in reduced ATP levels. Protein degradation pathways were also affected under Cr stress conditions, leading to the accumulation of truncated protein inside the cell. Under Cr (VI) stress, 26S-mediated proteolysis (via reduction in Rpn11 proteasome subunit) was affected, which impaired protein degradation [161]. Sharmin et al. [162] reported 36 differentially expressed proteins in *M. sinensis* under Cr stress conditions. Hence, it is pertinent to pay attention from the beginning of Cr exposure to cell interaction/receptor activation, associated signaling pathways, differential expression of related pathway genes/TFs, their metabolism, and thus the fate of these particles. Genetic engineering holds great promise for removing Cr from contaminated soils by engineered plants or tailored microbes. For instance, genetically engineering plants such as *Nicotiana tabacum*, *Populus angustifolia*, and *Silene cucubalis* to overexpress glutamylcysteine syntlietase resulted in increased heavy metal accumulation compared to wild type plants [163]. There have many success stories about genetically modified plants reducing different heavy metal contaminants in different environments. However, there are few studies on Cr contamination.

Synthetic biology has emerged as one of the most promising fields in modern agriculture to redesign current plant systems with more beneficial traits in terms of growth, yield, and stress adaptability. Many efforts have been made to discover plant species with more effective Cr detoxifying processes in certain locations. However, they may vary in their abilities to absorb, accumulate, and exclude Cr pollutants. These mechanisms are crucial since they will establish a plant’s specialized function in phytoremediation. In this context, synthetic biology can be used to engineer plants that have precise and extremely effective Cr mitigation capabilities for sustainable agriculture. Nevertheless, the most successful stories of synthetic biology have been reported with model microbes such as *E. coli* and yeast, which have helped to better understand an array of biological and environmental complex traits and further translate that knowledge in both plant and animal systems. In this review we focus on how synthetic biology can be employed in microbe- and plant-based Cr mitigation tools in sustainable agriculture. Improvement of Cr stress resilience in crops using the CRISPR/Cas system is the most advanced and efficient tool in modern agriculture. Applications of CRISPR/Cas-related technologies are presently being utilized to modify the genomes of multiple crop plants in order to withstand different biotic and abiotic stresses, while its expansion in heavy metal stress tolerance is still in the exploratory phase [164,165,166,167,168]. Nevertheless, use of the CRISPR/Cas system in Cr stress resilience in agricultural crops is a promising venture to accelerate their phytoremediation potential and other beneficial traits. Furthermore, the availability and accessibility of the whole genomic sequences of phytoremediators or models such as *Arabidopsis halleri*, *B. juncea*, *Hirschfeldia incana*, *Noccaea caerulescens,* and *Pteris vittata* could provide an excellent podium for the discovery and characterization of potential target key genes related to Cr uptake, signaling, translocation, and tolerance, providing further targets for CRISPR-based gene editing. According to Pérez-Palacios et al. [169], CRISPR/Cas systems can also promote a number of additional processes, including phytoaccumulation, phytostabilization, phytodegradation, and rhizofiltration, respectively. One of the most important targets for CRISPR-mediated Cr mitigation is its transporters since they aid in its uptake and translocation. Targeting these genes using CRISPR/Cas can dramatically improve the Cr detoxifying capacity of plants. Hence, there is need to expand the use of the CRISPR/Cas approach to engineer crops with improved Cr tolerance that can boost agricultural productivity and the economy.

Although microbes are essential to plant survival in Cr-contaminated soils, their effectiveness is constrained by shifting environmental factors, poor colonization, and limited permanence in the rhizosphere. In this regard, the application of tailored beneficial synthetic microbial communities (SynComs) using microbiome engineering will provide novel avenues for Cr mitigation in sustainable agriculture. In order to develop efficient tailored microbial consortia for Cr mitigation, there is a need to identify the elite microbial communities that can easily grow in Cr-contaminated soils. In addition, the identification and characterization of root exudates that influence the Cr microbial consortia are also important for developing tailored Cr SynComs. NGS technologies, multiomics, and computational methods have all significantly contributed to our understanding of how the microbiome community changes in response to biotic and abiotic stressors; however, little is known during heavy metal stress, especially Cr stress [170,171,172]. Therefore, it is crucial to investigate the microbiome of Cr-contaminated soils and find novel microbial species in order to create Cr SynComs that can provide novel mitigative alternatives for Cr removal in agricultural soils.

### 6.5. Breeding of Chromium-Safe Cultivars

Even though several remediation techniques and strategies have been used to mitigate the adverse effects of heavy metal toxicity in plants [173,174], none of these techniques has proven effective in terms of cost, time, and sustainability and in achieving the desirable results. Moreover, in recent years, the extent and adversity of the toxicity of chromium have gained wide attention worldwide. Hence, breeding programs aimed at developing crop varieties with high chromium resistance or tolerance are much needed to reduce the toxic effects of chromium in plants as well as in humans. Fortunately, a new concept has been proposed that includes the screening and developing of plant varieties with low chromium content in the edible parts. This concept originates from the fact that heavy metal accumulation differs not only among plant species but also among varieties of the same species [175]. The metal accumulation abilities varied among different species of crops such as garlic chives, carrots, cucumbers, tomatoes, and radishes [176,177]. Additionally, it varied among cultivars of the same species; for instance, high metal accumulation was found in sweet corn cultivars but not in normal corn cultivars [178]. Likewise, in *B. napus*, ZS 758 and Zheda 622 were low and high chromium-accumulating cultivars, respectively [179]. In *Oryza sativa*, Xiushui 113, Xiushui 09, and Mingzhu 1 were low Cr-accumulating cultivars whereas HG 5, Dan K5, and Huyou 1 were high Cr-accumulating cultivars [180]. In *Triticum aestivum*, Kohsar-95, Meiraj-08, Millet-011, C-217, and NARC-011 were low Cr-accumulating cultivars whereas Auqab-00 and Pakistan-13 were high Cr-accumulating cultivars [181]. Similarly, cultivars of staple crops such as wheat, maize, and rice have also depicted variations in the uptake of metals and since these staple crops form an inseparable part of the human diet across the globe, more attention has been paid to the development of crops with low metal uptake [182]. Plant varieties also differ in their maximum thresholds of Cr tolerance [183]. Nath et al. [184] reported the differential effects of Cr on the germination of rice seeds and the growth of seedlings. Not only chromium, but differential tolerance to cadmium has also been reported in many crops. This further substantiates the concept of screening and developing high metal-tolerant plants. The term pollution safe cultivars (PSCs) has been proposed for such plants and a strategy has been proposed for selecting such PSCs. PSCs are crops with metal accumulation in the edible parts to an extent that is safe to eat [178]. As per the WHO, the permitted concentration of hexavalent chromium in the edible part is 1.3 mg/kg. In the past, cadmium-safe cultivars have been screened in sunflower and durum wheat [175]. On the basis of metal concentrations and bioaccumulation factors, plants are classified as low, moderate, and high metal accumulators [185]. However, the ability to accumulate metals varies according to the environmental conditions and it is also necessary to consider cultivar–environment interactions when classifying plants as metal-tolerant or -sensitive. Jun et al. [186] also reported different chromium tolerance levels in several pulse crops. On the basis of germination and seedling growth, they concluded that *Lablab purpureus* and *Glycine max* were the most sensitive species whereas *Lathyrus odoratus* and *Dumasia villosa* were the most tolerant species. The selection of crop cultivars with high metal tolerance using different breeding approaches could provide an effective solution. Both conventional and modern breeding approaches may be employed to screen and develop chromium-tolerant cultivars. For instance, mutation breeding technique may be employed in different staple crops with the aim of isolating mutant lines with improved chromium tolerance, mutant lines with less uptake of chromium, or mutant lines that accumulate chromium within the cell walls, restricting its transfer into cells and then into trophic levels. However, conventional breeding approaches are arduous, time-consuming, and laborious, and therefore, modern breeding approaches such as marker-assisted selection may be used for the selection of metal tolerant cultivars [187]. For instance, Qui et al. [188] reported three QTLs, viz., qRCA7, qSCA9 and qSRA7, that were associated with Cr accumulation in the roots and shoots of rice. These markers may help in the selection of low Cr-accumulating cultivars of rice. Almas et al. [181] identified SNPs associated with chromium tolerance in spring wheat. They reported 71 marker trait associations (MTAs) distributed over 47 loci in Cr treatment and 43 MTAs distributed over 29 loci in control treatment. In China and the United States of America, breeding programs have achieved success in screening and developing low metal varieties of sunflower and durum wheat [182]. Breeding of low metal crops is an emerging and promising strategy for alleviating heavy metal toxicity. However, further research is needed to formulate the criteria for identifying stable, low metal-accumulating crops. In modern breeding approaches, more insight is needed to identify and regulate the expression of genes associated with metal uptake, accumulation, and exclusion.

## 7. Conclusion and Future Perspectives

Cr pollution is becoming a serious problem to the ecosystem and a major health risk to the biota. Additionally, the detrimental effects of Cr on our agriculture are a serious concern for food security and safety. Hence, there is a need to find a long-lasting remedial tool kit for its removal from the environment. This requires a deep understanding of Cr accumulation, translocation, and plant defense responses in both model and crop systems. In this review, we discussed Cr toxicity in plants, its signaling cascades, and various mitigation approaches. However, there are many questions about Cr perception and signal transduction that warrant future attention. For example, how cell wall receptor kinases and ion channels respond to Cr stress. How Cr triggers Ca^2+^ and ROS signaling cascades and how it modulates different intracellular signaling cascades in roots. The function of the many ion transporters that promote Cr transport as well as the several carriers that assist in its movement from the roots to various compartments require future consideration. Moreover, how Cr stress triggers hormonal activation or how it regulates cross talk can have different outcomes in terms of plant growth and defense. Further, we also highlighted the importance of various priming approaches for mitigating Cr toxicity. However, recent research suggests that plants can be primed by various methods, such as microbial, chemical, and nanotechnology-based methods, to better tolerate Cr stress. However, these tools have some limitations that need to be improved in order to attain their maximum efficiency. For instance, the unregulated deposition of chemical or nanoparticles during priming to increase plant protection, which affects soil fertility and crop output, significantly alters plant ecosystems and the microbiota. Although using beneficial microorganisms to prime plants rather than chemicals can address some of the issues with chemical priming, their high-efficiency formulations are dependent on a number of factors. In this context, future studies should focus on exploring the microbiome in Cr-contaminated soils and identify elite Cr-tolerant taxa that can be engineered in order to develop tailored Cr SynComs. Additionally, genetic engineering and advanced breeding tools, such as genome editing, can also provide the best approaches to develop long-lasting Cr-tolerant crop cultivars for sustainable agriculture.

## Figures and Tables

**Table 1:** Role of microbes in mitigating chromium toxicity by converting Cr (VI) to Cr (III).

Microorganisms	pH	Mechanisms	References
Bacteria			
*Serratia* sp. C8	6–8	Bioreduction	[46]
*Sphingopyxis macrogoltabida* SUK2c	7	Bioreduction, Biosorption	[47]
*Bacillus methylotrophicus*	7	Bioreduction	[48]
*Pisolithus* sp1	5–6	Bioreduction, Biosorption	[49]
*Sporosarcina saromensis* M52	7–8.5	Bioreduction	[50]
*Asperillus flavus* CR500	6.5	Bioreduction, Biosorption	[51]
*Leiotrametes flavida*	6	Biosorption	[52]
*Sporosarcina saromensis* M52	2	Biosorption	[53]
*Bacillus salmalaya*	3	Biosorption	[54]
*Enterobacter cloacae*		Biosorption	[55]
*Chelatococcus daeguensis*	7	Biosorption	[56]
*Micrococcus* spp.	7	Biosorption	[57]
*Planococcus* sp. VITP21	6.8	Biosorption	[58]
*Pseudomonas alcaliphila* NEWG-2	7	Biosorption	[59]
*Halomonas* sp. DK4	6	Biosorption	[60]
*Klebsiella* spp.	9	Biosorption	[61]
*Sinorhizobium* sp. SAR1	1	Biosorption	[62]
*Pseudomonas aeruginosa CCTCC AB93066*	7.0	Biosorption	[63]
*Bacillus cereus ZY-2009*	7.0	Bioreduction	[64]
Fungi			
*Paecilomyces lilacinus, Penicillium commune, Fusarium equiseti,* and *Cladosporium perangustum*	4	Biosorption	[65]
*Aspergillus versicolor*	6	Biosorption	[66]
Consortium of *Rhizopus oryzae, Aspergillus lentulus,* and *Aspergillus terreus*	6.5	Biosorption	[67]
*Aspergillus terreus*		Biosorption	[68]
Microalgae			
*Pseudanabaena mucicola*	2	Biosorption	[69]
*Chlorella colonials*		Biosorption	[70]
*Chlorella vulagris*	3	Biosorption	[71]
*Chlamydomonas* spp.	4	Biosorption	[72]

**Table 2:** Roles of chemical and nano-priming in mitigating Cr toxicity in different plants.

Name of Compound	Effect on Chromium Toxicity	Alleviated Physiological Effects under Chromium Toxicity	Crop Plant under Investigation	References
Chemicals Used for Alleviating Cr Toxicity				
Menadione sodium bisulfite (MSB)	Considerably reduces accumulation and transport	Reduces oxidative stress and membrane electrolyte leakage	Wheat	[116]
Melatonin (MT) (N-acetyl-5-methoxytryptamine)	Detoxification of Cr toxicity	Activates antioxidant system Helps plants in maintaining leaf water status through improvement of root structures	Maize	[143]
Taurine	Lesser accumulation of Cr in aerial parts of plants	Increases proline content Lowers aerial B and Cr levels Strengthens antioxidant defense systems Lowers the ROS levels Increases the synthesis hydrogen sulfide, nitric oxide, glutathione, and phenolic compounds	Wheat	[77]
Hydrogen sulfide	Restriction of uptake	Activation of the antioxidant system	Rice Wheat Barley	[107,122]
Indole acetic acid	Restriction of uptake	Improves various growth and developmental traits, such as root and shoot length, fresh weight Activates antioxidant system, increases photosynthetic pigments	Rice	[139]
Brassinosteroid	Decreases Cr-induced phytotoxicity by lowering Cr uptake, accumulation, and translocation	Stimulates antioxidative defense systems Photosynthetic attributes Improves seed germination, plant growth, and biomass	Soybean	[101]
Sodium nitroprusside (SNP)	Restriction of uptake	Increases biomass and water potential Enhances antioxidant system activity	Maize	[144]
Glutathione	Increases Cr accumulation Improves Cr tolerance Decreases Cr toxicity	Increases antioxidant activity and photosynthesis pigments Increases osmolytes Increases the expression of genes related to alleviation of Cr toxicity physiological adaptability	Soybean	[109]
Glycine betaine	Reduces accumulation of Cr	Significantly enhances plant growth and yield Enhances biochemical and physiological traits (antioxidant enzyme activities)	Chickpea	[145]
Hydrogen peroxide (H_2_O_2_)		Inhibits cell death Decreases the accumulation of Cr in roots Stimulates sulfur assimilation Enhances antioxidants and proline metabolism	WheatRice	[99,146]
Citric acid chelate	Reduces accumulation of Cr	Improves germination, growth, and yield Increases activities of antioxidant enzymes such as SOD, POD, CAT, and APX Enhances accumulation of non-enzymatic antioxidant molecules	Wheat	[147]
Iron (Fe)–lysine (lys)	Reduces accumulation of Cr	Helps in improving plant growth and composition Decreases the concentrations of ROS and Cr	Rapeseed	[148]
Nitric oxide (NO)	Reduces uptake and accumulation of Cr in roots	Inhibits death of cells Lowers accumulation of Cr in roots Higher assimilation of sulfur Enhances antioxidants Enhances proline metabolism	Wheat	[146]
Nanoparticles for Alleviating Cr Toxicity				
SiNPs	Reduces the uptake and accumulation of Cr	Modulates a number of biochemical, physiological traits related to growth and stress resilience Increases root and shoot length and fresh weight Increases the content of antioxidant and osmoprotectants	RicePea	[124,139]
Cerium dioxide nanoparticles (CeO2)	Reduces the uptake and accumulation of Cr^6+^ and Cr^3+^	Increases plant biomass and growth Alleviates oxidative stress Improves antioxidant enzymatic functions	Sunflower plants	[149]
Fe nanoparticles (Fe NPs)	Reduces the uptake and accumulation of Cr	Reduces Cr-induced oxidative damage Enhances non-enzymatic and antioxidant activity Increases growth and yield traits Increases photosynthetic activity	Rice	[150]
Zinc oxide nanoparticles (ZnO NPs)	Detoxification of Cr	Induces activity of antioxidative enzymes Leads to overexpression of antioxidative genes Improves efficiency of photosynthesis	Wheat Rice	[141,151]
Green copper nanoparticles	Immobilizes Cr in the soil	Augments plant growth, biomass, and cellular Enhances antioxidants contents Decreases the reactive oxygen species	Wheat	[152]
Citrate-coated magnetite nanoparticles (NPs)	Diminishes the toxicity effects of Cr	Enhances seed germination Enhances growth of roots and shoots Lowers accumulation of heavy metal	Wheat	[153]
Nano-zerovalent iron Nanoparticles	Decreases Cr uptake and buildup	Improves shoot and root dry weight Increases photosynthetic pigments such as chlorophyll and carotenoids Increases proline content Improves efficiency of cell redox homeostasis	Sunflower	[154]
Metallic nanoparticles	Reduces the uptake and toxicity of Cr	Increases photosynthetic activity Regulation of cellular metabolites Increases chelation capacity to bind with Cr Enhances the activity of antioxidants Helps in reducing Cr-induced oxidative stress and cellular damage Detoxification of Cr	Rapeseed Rice	[155]

## Data Availability

Not applicable.
